# Validity and Reliability of Kinematics Measured with PUSH Band vs. Linear Encoder in Bench Press and Push-Ups

**DOI:** 10.3390/sports7090207

**Published:** 2019-09-10

**Authors:** Roland van den Tillaar, Nick Ball

**Affiliations:** 1 Department of Sport Sciences and Physical Education, Nord University, 7601 Levanger, Norway; 2 Faculty of Health, Research Institute for Sport and Exercise Science, University of Canberra, Canberra 2601, Australia

**Keywords:** peak velocity, mean velocity, resistance training, strength, 1 RM

## Abstract

Background: The aim of this study was to compare the validity and reliability of a PUSH band device with a linear encoder to measure movement velocity with different loads during the push-up and bench press exercises. Methods: Twenty resistance-trained athletes performed push-up and bench press exercises with four different loads: without weight vest, 10-20-30 kg weight vest, bench press: 50–82% of their assumed 1 repetition maximum (1 RM) in steps of 10 kg. A linear encoder (Musclelab) and the PUSH band measured mean and peak velocity during both exercises. Several statistical analyses were used to investigate the validity and reliability of the PUSH band with the linear encoder. Results: The main findings of this study demonstrated only moderate associations between the PUSH band and linear encoder for mean velocity (r = 0.62, 0.70) and peak velocity (r = 0.46, 0.49) for both exercises. Furthermore, a good level of agreement (peak velocity: ICC = 0.60, 0.64; mean velocity: ICC = 0.77, 0.78) was observed between the two measurement devices. However, a significant bias was found with lower velocity values measured with the PUSH band in both exercises. In the push-up, both the linear encoder and PUSH band were deemed very reliable (ICC > 0.98; the coefficient of variation (CV): 5.9–7.3%). Bench press reliability decreased for the PUSH band (ICC < 0.95), and the coefficient of variance increased to (12.8–13.3%) for the velocity measures. Calculated 1 RM with the two devices was the same for the push-up, while in bench press the PUSH band under-estimated the 1 RM by 14 kg compared to the linear encoder. Conclusions: It was concluded that the PUSH band will show decreased reliability from velocity measures in a bench press exercise and underestimate load-velocity based 1 RM predictions. For training, the PUSH band can be used during push-ups, however caution is suggested when using the device for the purposes of feedback in bench press at increasing loads.

## 1. Introduction

Quantifying and prescribing training intensity objectively from strength training programs is a challenge [1,2]. Typically, training intensity is prescribed as a percentage of the maximal intensity that the athlete can perform in that exercise and is considered a fundamental variable for the design of strength training programs [1]. Training intensity is usually based on the individuals’ one repetition maximum (1 RM: the maximum load that can be lifted once). However, this requires performing a maximal lift, which may have contra-indications due to the highly intensive effort, the chance of failure, and the achieved value changing over several weeks of the program [3].

Over the past few years, the introduction of velocity-based feedback to quantify training loads for strength training exercises has allowed more instantaneous modification of loading to accommodate strength changes [4,5,6,7]. This is based on the well-known load-velocity relationship [8,9,10], for which lighter loads are moved at faster velocities and heavier loads are moved at slower velocities. This method has been applied to numerous exercises, with barbell velocity during the bench press and back squat shown to be highly correlated with relative 1 RM training intensity over a wide range of loads [8,9,10,11,12,13]. Thus, the method for monitoring strength training intensities using barbell velocity is a valid approach to training intensity prescription.

When basing training intensity on velocity, it is imperative that the system used to measure velocity be both valid and reliable. The commercial market has many systems that allow a movement velocity to be measured for such purposes. Among these systems, the most widely used technology to track barbell velocity are linear encoders. These encoders consist of a sensor with a cable that is attached to a barbell, which measure barbell velocity by differentiating cable displacement with respect to time (i.e., linear position encoder) [14] or by recording electrical signals proportional to cable velocity (i.e., linear velocity encoders) [15]. These are used because of their accuracy and relative ease of use [11,14,15]. However, they are expensive and potentially less user friendly for the average athlete, which limits their use outside professional sport settings or the laboratory.

In recent years, inertial measurement units (IMU), that are paired with a smartphone application to transfer data through Bluetooth or Wi-Fi connections, have gained prominence to measure movement velocity in resistance exercises by integrating the acceleration data with respect to time [16]. These IMUs are small and wireless connected to a smartphone, easier to take with you for training and much cheaper than an average linear encoder system. Therefore, more accessible for an average athlete that wants to monitor his/her training intensity and performance at different loads. One of these systems is the PUSH band, which is an IMU that is connected to a smartphone and gives mean and peak velocity after each repetition. To date, some studies have investigated reliability and validity of the PUSH band with three of these focusing on the bench press exercise [2,9,17,18]. Orange et al. [18] concluded that the PUSH band gave no highly valid and reliable mean and peak velocities in comparison to a linear encoder at 20, 40, 60, 80 and 90% of 1 RM. Whereas Perez-Castilla et al. [19] and McGrath et al. [17] indicated that the PUSH band provided highly reliable and valid measurements of mean velocity when the data of several loads were combined. However, Perez-Castilla et al. [19] showed that the PUSH band seems to be compromised when the data of individual loads were analyzed separately, with increasing load the mean velocity was more underestimated with the PUSH band. McGrath et al. [17] found that the PUSH band underestimated mean velocity at both low and high loads (40 and 80% of 1 RM). As indicated previously by Banyard et al. [9] and Perez-Castilla et al. [19], the validity and reliability of the device may be impacted by the load used, therefore, further assessment of the PUSH band device to accurately measure velocity in upper body exercises at different loads is warranted. Furthermore, the placement of the PUSH band could have an influence on the discrepancy of the mean and peak velocity measurements with the linear encoder.

The push-up exercise is a similar upper body exercise that have similar movement kinematics and targets the same muscles as in bench press [20] and are frequently used within training programs for upper body development. The main difference is that the hands are fixed on the ground and the own body with eventual extra added (weight vest) are used in the push-ups, in bench press the load is moved while lying supine on a bench. Thereby the placement of the PUSH band differs: around the upper arm during the push-up exercise and around the forearm distal to the elbow in the bench press. These differences in placement could have an influence on the measurements compared with a linear encoder during both exercises. Therefore, the aim of this study was to compare the validity and reliability of PUSH band with a linear encoder to measure movement velocity with different loads during push-up and bench press exercises.

## 2. Materials and Methods

The criterion validity and reliability of mean and peak velocity measurements generated from the PUSH Inc. (PUSH Inc., Toronto, ON, Canada). PUSH band was assessed in a bench press and push-up exercise. Mean and peak velocity was simultaneously collected from a linear encoder, which was used as the reference values to assess the PUSH band’s performance. The velocity values from the two exercises across four different loads was used to see the impact of load and exercise type on the outcome measures. Participants completed three repetitions at each load. 

### 2.1. Subjects

Twenty resistance-trained athletes (age 22.5 ± 5.24 years, body mass 83.7 ± 10.7 kg, body height 1.80 ± 0.06 m with at least two years of at least two times per week of resistance training experience) participated in the study. The participants were instructed to avoid undertaking any additional resistance training targeting the upper body during the 72 h prior to testing. Each participant was informed of the testing procedures and possible risks, and a written consent was obtained prior to the study. The study complied with the current ethical regulations for research and approved by the Norwegian Center for Research Data (number: 42440), and conformed to the latest revision of the Declaration of Helsinki.

### 2.2. Procedures

After a general warm-up of 5 min treadmill jogging, the participants performed sub-maximal attempts (three repetitions) with different loads in both bench press and push-up (with a weight vest: 10–30 kg) to familiarize themselves with the exercise requirements. The participants were then randomly split into two groups. One group performed the push-up first while the other group started with the bench press condition. The participants performed each condition (push-up and bench press) with four different loads; Push-up: without weight vest, and then with a weight vest weighing 10 kg, 20 kg and 30 kg weight vest. For the bench press four loads starting at 50% of their self-estimated 1 RM (based upon their experience from the last half year of training) and incrementing in steps of 10 kg was performed. This equated to 50–82% of the individuals’ 1 RM. No 1 RM was tested to avoid the chance of injury. Furthermore, due to the purpose of the study not necessary since the aim was to investigate reliability and validity of the PUSH band at different regularly used training loads on which often 1 RM is estimated upon by linear regression [7]. Three repetitions per load were performed and the load order was randomized. In the familiarization warm-up, each participant used their preferred grip width in bench press, which was then subsequently standardized for each condition and load.

### 2.3. Measurements

**Figure 1:**
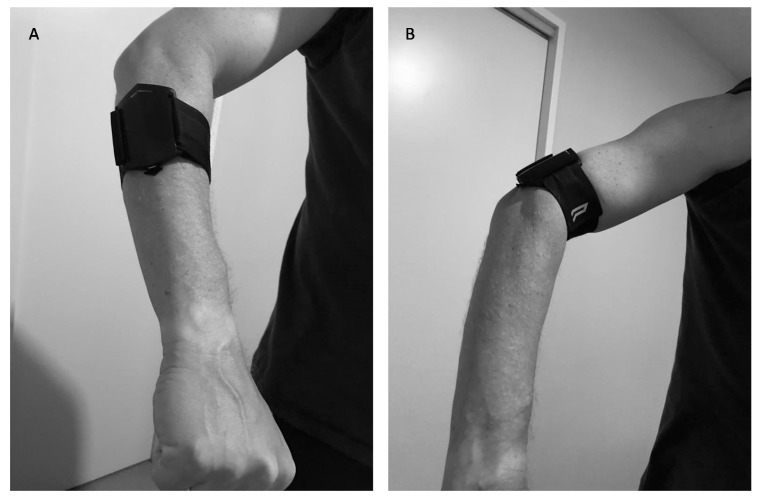
Placement of the PUSH band on the arm during the (**A**) bench press and (**B**) push-up.

The PUSH band is a smartphone-based wearable device designed to track movement velocity during a variety of resistance exercises. The PUSH band consists of a 3-axis accelerometer and a gyroscope that provides six degrees of freedom in its coordinate system sampling at 200 Hz. Prior to the test the PUSH band was attached to the arm according to the specifications of the manufacturer. In the push-up, the PUSH band was placed around the upper arm. In the bench press, the band was placed around the forearm distal to the elbow (Figure 1A,B). Participants were instructed to not move or adjust the band during the study. To process the PUSH band data, a Butterworth filter was used to smooth the acceleration data, and vertical velocity was calculated by the integration of the vertical acceleration with respect to time. Readers are referred to Balsalobre-Fernandez et al. [2] for further information on the specific calculation methods. The PUSH band displays mean and peak velocity during the concentric phase of the push-up and bench press. The PUSH band was linked to an iPhone PUSH app. v.1.10.4 using a Bluetooth 4.0 LE connection to record the measured data.

A linear encoder (ET-Enc-02, Ergotest Technology AS, Porsgrunn, Norway) was attached to the barbell when performing bench presses and attached loosely around the sternocostal region using a collar when performing a push-up. The linear encoder measured with a resolution of 0.019 mm and counts the pulses with a 5 msinterval during vertical displacement in relation to the lowest point of the barbell (zero distance). Peak and mean upward velocity was calculated by using a five point differential filter with with Musclelab^TM^ v10.73 software (Ergotest Technology AS, Porsgrunn, Norway). Bosquet et al. [15] have shown previously that the linear encoder used in this study produced highly valid and reliable measurements in bench press testing (r = 0.93) and was therefore used as the criterion in this study.

Using the mean and peak velocities from each device at each different load, a load-velocity relationship was established as a product of the load and mean velocity over three repetitions for each participant. Based on the athlete’s performance with the various loads, a linear regression was applied to calculate the theoretical 1 RM for each participant. The x-variable was set as 0.2 m/s, which indicates the velocity where a 1 RM theoretically is attainable [11]. To calculate 1 RM the following formula were used:

𝑦 = 𝑎 × 0.2 𝑚/𝑠 + 𝑏(1)

Both the coefficient of x (a), and y-intercept (b) is individual for each subject. To establish *a* and *b* in the linear equation for each participant, a scatter plot with an added linear regression line was calculated in Excel. By replacing x (a) with 0.2 m/s in the formula, the load-velocity relationship for maximal performance was established for each participant and theoretical 1 RM could be calculated.

### 2.4. Statistical Analyses

To compare the validity and reliability of the two measuring devices in bench press and the push-up, several statistical analyses were used similar to the equipment validation study of Balsalobre-Fernandez et al. [2]. First, to analyse the validity of the PUSH band, using Pearson’s product-moment correlation coefficient (Pearson’s *r*) was used for both peak and mean velocity in comparison with the linear encoder. The criteria to interpret the strength of the r coefficients were as follows: trivial (<0.1), small (0.1–0.3), moderate (0.3–0.5), high (0.5–0.7), very high (0.7–0.9), or practically perfect (>0.9) [21]. Secondly, the standard error of estimate (SEE) was used to assess the typical error in the measurements and Bland–Altman plots to identify potential systematic bias, which were reported through mean-bias and standard deviations. Thirdly, to assess reliability of the three repetitions of each set with both the PUSH band and the linear encoder the ICC (3,1), the coefficient of variation (CV), and test–retest correlations (r) were used. The thresholds for interpreting ICC results were: 0.20–0.49 low, 0.50–0.74 moderate, 0.75–0.89 high, 0.90–0.98 very high and ≥0.99 extremely high [21]. The average reliability of each measure was interpreted as acceptable for an ICC ≥ 0.70 and a CV ≤ 10%, moderate when ICC < 0.70 or CV > 10%, and unacceptable/poor when ICC < 0.70 and CV > 10% [22]. A two-way ANOVA (weight and measuring device) with repeated measurement was used for the peak and mean velocity in both bench press and push-up exercises. Where the sphericity assumption was violated the Greenhouse-Geisser-corrected *p*-values in the results were reported. Post hoc tests using the Holm-Bonferroni probability adjustment were used to identify differences. In addition, a one-way ANOVA with repeated measures (weight) was performed to identify if reliability (CV) over the three repetitions changed by weight or measuring device. The effect size used and reported in this study was partial eta squared (η^2^), where 0.01 ≤ η^2^ < 0.06 constituted a small effect, 0.06 ≤ η^2^ < 0.14 constituted a medium effect, whereas η^2^ < 0.14 constituted a large effect [23]. Finally, linear regressions were used to analyse the load-velocity relationship and to identify predicted 1 RM of each subject in both exercises with each measuring device. The level of significance was set at *p* ≤ 0.05 for all tests and the analyses were carried out with SPSS Statistics v25 (SPSS Inc., Chicago, IL, USA) was used.

## 3. Results

**Figure 2:**
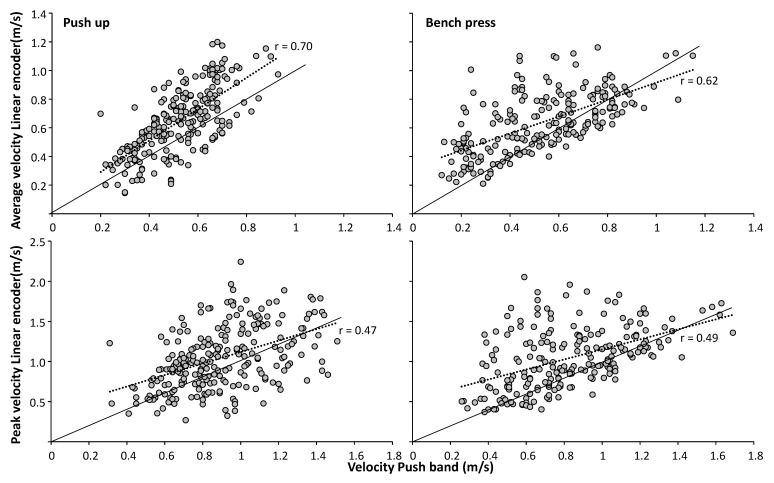
Correlation between PUSH band and linear encoder for mean and peak velocity during the push-up and bench press for all 240 repetitions measured. Solid black line indicates identical results between the two measuring devices.

**Figure 3:**
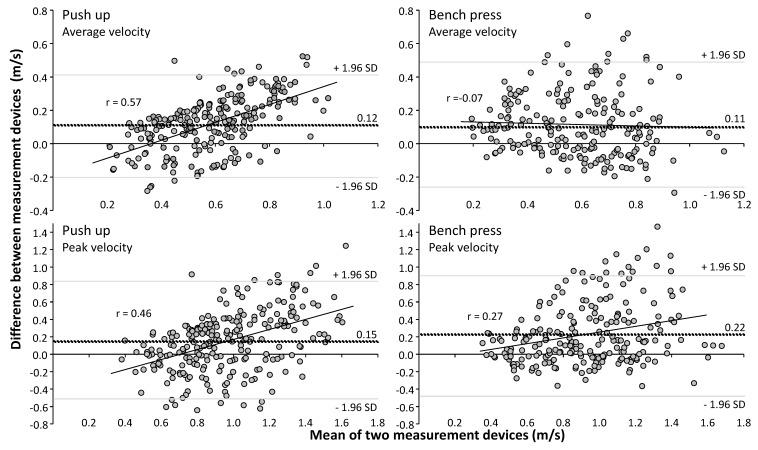
Bland-Altman plots between PUSH band and linear encoder measured peak and mean velocity in bench press and push-up. Dash line indicates a systematic bias between the two measuring devices (positive values mean higher velocity obtained with the linear encoder than the PUSH band). The grey lines represent 95% Confidence intervals.

Across all load conditions and exercises, correlations revealed significant (*p* < 0.001) low to moderate associations between the PUSH band and linear encoder (Figure 2) for mean velocity (r = 0.70, SEE = 0.16 and r = 0.62, SEE = 0.17 for push-up and bench press) and peak velocity (r = 0.46, SEE = 0.34 and r = 0.49, SEE = 0.33 for push-up and bench press). A systematic bias between the two measuring devices was found in both exercises for the mean (0.11 and 0.12 m/s) and peak velocity (0.15 and 0.22 m/s) where the linear encoder measures were significantly higher than the PUSH band (Figure 3). 

Reliability was different between the exercises. Across the three repetitions within the push-up both the linear encoder and PUSH band were very reliable and acceptable with ICCs of 0.98, CV of 5.9–7.3% and test-retest correlations of 0.94–0.95. However, within the bench press exercise, whilst the linear encoder measures showed the same reliability as the push-up (ICC = 0.98), the PUSH band reliability decreased to moderate (ICCs = 0.95 and 0.92, CV of 12.8–13.3% and test-retest correlations of 0.81 and 0.87, Table 1) although. 

**Figure 4:**
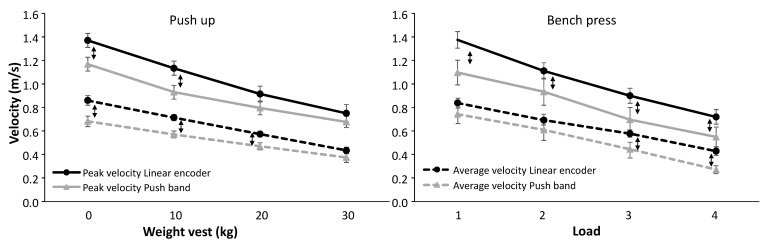
Mean (± average standard deviation over three attempts per subject) and peak velocity measured with the PUSH band and linear encoder during the Push-up and bench press with four different weights. ↕ indicates a significant difference between the two measurement devices with this weight.

A two-way ANOVA on the mean and peak velocity calculated over the three repetitions, showed that besides the increase in velocity based on the weight lifted (F ≥ 142, *p* ≤ 0.001, η^2^ ≥ 0.89), an effect was found for the measuring devices (F ≥ 7.5, *p* ≤ 0.014, η^2^ ≥ 0.31, Figure 4) except the peak velocity in the push-up (F = 4.0, *p* = 0.059, η^2^ = 0.18). In addition, an interaction effect (weight*measuring device) for all variables was found (F ≥ 3.1, *p* ≤ 0.037, η^2^ ≥ 0.16) indicating a difference in the increases of mean and peak velocity as weight increased between the two measuring devices. Post hoc comparison revealed that with the push-up, peak and mean velocity were significantly higher for the linear encoder when performing without extra weight and with 10 and 20 kg weight vest measured compared to the PUSH band. In bench press for the peak velocity the linear encoder measured significantly higher with all loads compared with the PUSH band, while for the mean velocity this was only found with the two heaviest loads (Figure 4).

**Figure 5:**
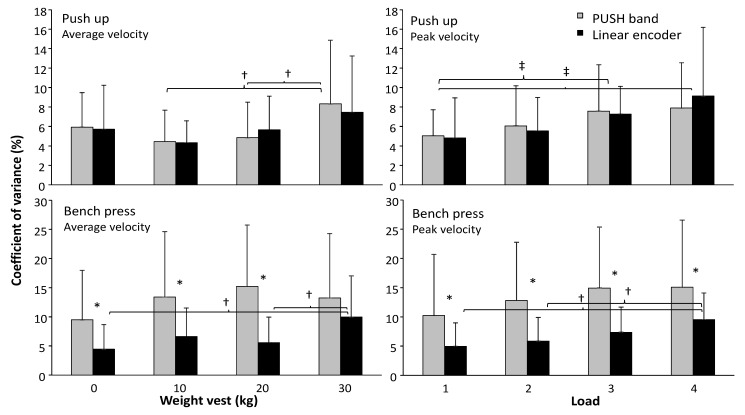
(± standard deviation) coefficient of variance of the average and peak velocity measured with the PUSH band and linear encoder during the Push-up and bench press at each of the four different weights. * indicates a significant difference between the two measurement devices with this weight.

When comparing the coefficient of variation for each load for the peak and mean velocity in both exercises measured with the PUSH band and the linear encoder a significant effect was found for weight in both mean (*p* = 0.008) and peak velocity (*p* = 0.004) during the push-up and in peak velocity during the bench press exercise (*p* = 0.022). An effect of measuring device was found only in bench press for both variables (*p* < 0.01) with no further significant interaction effects. Post hoc comparison revealed that CV of mean and peak velocity were significantly higher with almost all loads in bench press (Figure 5) when measured with the PUSH band compared with the linear encoder (except the heaviest load in mean velocity). In bench press the CV increased only significantly with the linear encoder when measuring with the heaviest weights. For the push-up exercise the CV increased with the mean velocity with the heaviest weight when measured with the PUSH band, while the CV during the peak velocities with the lowest weight was significantly lower than with the two heaviest weights for both measurement devices (Figure 5). 

Very good similarity between the load-velocity relationships from each measurement device based on the mean velocity from the four different loads in push-ups and bench press (r = 0.98 ± 0.02). However, the calculated 1 RM was significantly lower in bench press when measured with the PUSH band (86.8 ± 15.6) compared with the linear encoder (101.1 ± 27.5), while no significant differences in calculated 1 RM was found for the push-up (51 ± 16.4: PUSH band vs. 47.3 ± 10.7kg: Linear encoder).

## 4. Discussion

The main findings of this study demonstrated only low to moderate associations between the PUSH band and linear encoder for mean velocity (r = 0.62, 0.70) and peak velocity (r = 0.46, 0.49) for both exercises. A significant bias was found with lower velocity values measured with the PUSH band in both exercises. In the push-up, both the linear encoder and PUSH band were very reliable, but in bench press reliability decreased for the PUSH band, especially the CV which increased to 12.8–13.3%. Calculated 1 RM with the two devices was the same for the push-up, while in bench press the PUSH band estimated the 1 RM around 14 kg lower than with the linear encoder. 

A closer view at the correlations between the two measuring devices revealed that the PUSH band recorded lower velocities at most loads compared to the linear encoder, and that at only very light loads (mean velocity > 0.8 m/s and peak velocity > 1.4 m/s in bench press) velocity measures were greater from the PUSH band (Figure 2). These low-velocity loads would typically occur at 50% 1 RM for a bench press [11]. The current study also showed that the differences in velocity between the two measurement devices increases during push-ups when measuring high velocities as indicated by the positive correlations (r = 0.46 and r = 0.57 for resp. peak and mean velocity) and visible in the Bland-Altman plots (Figure 3). Thus, at higher velocity movements in the push-up, the PUSH band recorded lower values compared to the linear encoder, which was in disagreement with prior studies when looking at bench press [2,9]. An explanation for this may be the prescribed placement of the PUSH band. In the present study, the band was placed on the upper arm or forearm, which alongside the expected up and downwards motion associated with the exercises, will also causes a joint rotation about the longitudinal axis of the segment.

During the squats this rotation did not occur and thereby the whole acceleration of the PUSH-band was in a predominantly vertical direction similar to the linear encoder. This is shown in Figure 4, whereby push-ups performed with no extra load or with 10 and 20 kg weight vests caused significantly lower mean and peak velocities with the PUSH band compared to the linear encoder. During a bench press, peak velocity per load was always measured lower with the PUSH band and for mean velocity this was only significantly lower with the heavier loads (Figure 4). This findings suggest that at different loads and in different upper body exercises that the PUSH band and linear encoder are not interchangeable as velocity monitoring devices

Due to the different readings only occurring at the lower loads in push-ups between the two devices, the calculated 1 RM was not significantly different. In bench press the opposite was found, whereby at higher loads the differences in mean velocity increased between the two devices, resulting in a significantly higher calculated 1 RM for the linear encoder, compared with the PUSH band (Figure 4). This further indicates that the PUSH band and linear encoder are not interchangeable when assessing in session velocity for the purposes of load prescription.

The reliability (CV%) of each measuring device for the push-up was 6.6–7.3% which is in accordance with earlier studies in squats and biceps curl with the PUSH band [2,9,24]. The CV% increased to 8% at higher loads, especially when performing push-ups with 30kg weight vests. Similar findings were found by Banyard et al. [9] who showed that at higher load levels CV increased for the PUSH band. The reasoning for this may be related to the experience of the participants. Most participants had not trained many push-ups with a weight-vest at increasing loads, thereby a learning effect could occur, which is a limitation of the study. In bench press the CV was much higher for the PUSH band (~13%) vs. the linear encoder (~7%) indicating a larger variation compared to the linear encoder for the same action. This was also found by Perez-Castilla et al. [19] who showed that in bench press the CV increased from 5 to 19% with loads from 45 to 85% of 1 RM when measured with the PUSH band. An explanation for this could be placement of PUSH band, which was different between the two exercises. During push-ups the PUSH band was around the upper arm, while during bench press the PUSH band was on the proximal part of the forearm (Figure 1). The bench press is an open kinetic chain exercise, which means that the distal segment is free to move, while the push-up is a closed kinetic chain exercise and the distal segments are fixed to the push up handles. This increases the degrees of freedom in the bench press [25] which could result in more movement variability and thereby increase the CV of the PUSH band. Furthermore the movement trajectory during a push-up is straight up and down, while in the bench press the barbell also has horizontal movement [26], which also could result in different trajectories and thereby increase variability and CV of the PUSH band in comparison to the linear encoder.

## 5. Conclusions

Based upon the findings of the present study it can be concluded that the PUSH band should not be used interchangeably with a linear encoder. In general, the PUSH band measures lower peak and mean velocities in both exercises than the linear encoder. Both measurement devices, show similar reliable and validity during push-ups, but in bench press the reliability decreases for the PUSH band to levels which are outside the scope required for monitoring training improvements. Furthermore, predicted 1 RM based upon the load-velocity relationships measured by the PUSH band and linear encoders are not interchangeable in bench press.

Based upon the findings of the present study we can conclude that the PUSH band and linear encoder do not result in comparable results of peak and mean velocity during push-up and bench press exercises and therefore not interchangeable during training of these exercises. The PUSH band can be used during push-ups, however caution is suggested when using the device for the purposes of feedback in bench press at increasing loads.

## Figures and Tables

**Table 1:** ICC, 95% confidence interval (CI, Coefficient of variance (CV) and test-retest reliability (r) for the linear encoder and PUSH band in bench press and push-up when comparing peak velocity and mean velocity of three repetitions of each set.

Variable	ICC	CI	CV	R
Push-Up				
Linear encoder				
Peak velocity	0.98	0.97–0.99	7.3 ± 3.0%	0.95
Mean velocity	0.98	0.97–0.99	5.9 ± 1.7%	0.95
PUSH band				
Peak velocity	0.98	0.97–0.99	6.6 ± 1.3%	0.94
Mean velocity	0.98	0.97–0.99	6.6 ± 1.3%	0.95
Bench Press				
Linear encoder				
Peak velocity	0.98	0.98–0.99	6.9 ± 2.0%	0.96
Mean velocity	0.98	0.97–0.99	6.6±2.4%	0.96
PUSH band				
Peak velocity	0.92	0.89–0.95	13.3 ± 2.3%	0.81
Mean velocity	0.95	0.93–9.97	12.8 ± 2.4%	0.87
